# Pregnancy-Associated Plasma Protein-A2 and Anthropometry, Lifestyle, and Biochemical Factors in a Human Adult Population

**DOI:** 10.1038/s41598-017-10629-y

**Published:** 2017-09-05

**Authors:** Astrid Steinbrecher, Jürgen Janke, Matthew N. Poy, Claus Oxvig, Tobias Pischon

**Affiliations:** 1 Molecular Epidemiology Group, Max Delbruck Center for Molecular Medicine in the Helmholtz Association (MDC), Berlin, Germany; 2 Max Delbruck Center for Molecular Medicine in the Helmholtz Association (MDC), Berlin, Germany; 3 Department of Molecular Biology and Genetics, Aarhus University, Aarhus, Denmark; 4 Charité – Universitätsmedizin Berlin, Berlin, Germany; 5 MDC/BIH Biobank, Max Delbruck Center for Molecular Medicine in the Helmholtz Association (MDC) and Berlin Institute of Health (BIH), Berlin, Germany; 6 German Center for Cardiovascular Research (DZHK), partner site Berlin, Berlin, Germany

## Abstract

Pregnancy-associated plasma protein-A2 (PAPP-A2), a metalloproteinase purportedly related to pregnancy, foetal growth and development, has recently been described essential for pre-adult growth. Thus, we measured PAPP-A2 in plasma of a non-pregnant population and determined its associations with lifestyle, anthropometric or biochemical factors. In this cross-sectional study of 387 participants (20–70 years) randomly drawn from registration offices near Berlin, Germany, socio-economic and lifestyle factors were assessed by questionnaires, and anthropometric measures and blood samples were taken by trained personnel. Blood was analysed for standard clinical parameters. PAPP-A2 concentration was measured by ELISA. Generalized linear models were used to estimate associations with anthropometric and biochemical factors adjusted for age, sex, and weight. Adjusted mean PAPP-A2 concentration was slightly higher in women (283 pg/mL) than in men (261 pg/mL, p = 0.05) and positively correlated with age (r = 0.17, p = 0.001). PAPP-A2 concentration was inversely associated with body mass index (−2.7 pg/mL per kg/m^2^, p = 0.03) and weight (−1.0 pg/mL per kg, p = 0.01) and positively associated with γ-glutamyl transferase (13.6 pg/mL per SD, p = 0.02), aspartate transaminase (18.5 pg/mL per SD, p = 0.002) and lactate dehydrogenase (14.9 pg/mL per SD, p = 0.02). Our results support that PAPP-A2, beyond its established role in early growth and development is relevant in adult metabolisms.

## Introduction

Pregnancy-associated plasma protein-A and -A2 (PAPP-A2) are the only two members of the pappalysin family of metalloproteinases^1^. Substrates of both proteinases are subsets of the insulin-like growth factor binding proteins (IGFBPs); PAPP-A2 cleaves and inactivates IGFBP-3 and IGFBP-5^2^. Initial studies suggested that this proteinase primarily plays a role during pregnancy, since it is highly expressed in the placenta^2^, and circulating concentrations of PAPP-A2 were found to be increased during gestation^3^. Cleavage of IGFBPs is believed to cause increased insulin-like growth factor (IGF) bioavailability and bioactivity^4^, which is essential for foetal growth and development^5^.

While the first studies conducted on PAPP-A2 among humans focused on its role in pregnancy^3^ and in the context of maternal preeclampsia^6, 7^, *PAPPA2* expression is neither limited to the placenta nor to the blood during pregnancy^2^. Genetic data have pointed at PAPP-A2 as a determinant of human height^8^, and it was recently shown that the absence of PAPP-A2 in humans causes short stature^9^. However, to date, details on the role of PAPP-A2 in adult general populations remain unknown. The aim of this study was to measure PAPP-A2 in blood plasma of an adult human population and to evaluate whether anthropometric or lifestyle factors or biochemical parameters are associated with plasma concentrations of PAPP-A2.

## Subjects and Methods

### Study population

From September 2011 until December 2012, two cross-sectional pretest studies were undertaken at the study center (Max Delbruck Center, Berlin, Germany) to support the implementation of the German National Cohort^10^. For each pretest, a random sample of the general population aged 20–70 years living in the vicinity of the study center was drawn from the registration office. The first pretest also allowed the inclusion of participants through convenience sampling (e.g., via e-mail lists). Besides having the principal residence in the recruitment area, German language skills and the ability to give informed consent were eligibility criteria. All potential participants were invited to take part in a three hour examination at the study center, consisting of an interview, medical examinations and a blood draw.

All participants gave written informed consent. The study protocol was approved by the ethics committee of Charité University Medicine Berlin and by the local data protection officer. All examinations were carried out in accordance with the relevant guidelines and regulations.

### Data Assessment

Information on demography, lifestyle factors and medical conditions were collected in face-to-face computer-assisted interviews or via self-reported computer-assisted questionnaires. Participants indicated sex, date of birth, smoking status (never, former, current), frequency of alcohol consumption during the last 12 month (never or occasionally, 1–4 times/month, ≥2 times/week), and if they had ever been diagnosed with diabetes, osteoporosis, autoimmune diseases (i.e. systemic lupus erythematosus or Sjögren’s syndrome), or inflammatory bowel diseases (i.e. Crohn’s disease or ulcerative colitis) by a physician. Women were asked for reproductive details including current pregnancies and menstrual cycles (yes, no, don’t know).

Anthropometric measurements were performed by trained personnel according to standardized procedures. Wearing light underwear, participant’s weight in kg and height in cm was recorded on a digital stadiometer (SECA 285, SECA, Hamburg, Germany) up to one decimal place. Waist and hip circumferences were measured with a tape measure (SECA 201, SECA, Hamburg, Germany) in cm up to one decimal place.

Blood samples were collected in Sarstedt Monovette® tubes (2.6 mL serum-gel/clotting activator, 9 mL EDTA plasma, 2.7 mL EDTA plasma) following a standardized venipuncture protocol. Fasting status was not a prerequisite for blood draw.

The serum tubes were turned twice and rested at room temperature for 30–45 minutes before being centrifuged at 2000 g for 15 minutes at 15 °C. The EDTA plasma tubes were turned twice and set on a universal rocking mixer for a maximum of 5 minutes. The 2.6 mL serum-gel tube and the 2.7 mL EDTA plasma tube were shipped within 6 h to a laboratory for clinical diagnostics (Hospital Laborverbund Brandenburg-Berlin) for standard measurements of creatinine, uric acid, triglycerides, total cholesterol, high-density lipoprotein cholesterol (HDL-C), low-density lipoprotein cholesterol (LDL-C) (direct measurement), glycated haemoglobin A1 (HbA_1c_), sodium, potassium, γ-glutamyl transferase (GGT), aspartate transaminase (ASAT), alanine aminotransferase (ALAT), and lactate dehydrogenase (LDH), haemoglobin, haematocrit, erythrocytes, and leukocytes. Creatinine concentration was used to estimate glomerular filtration rate (eGFR) in mL/minute according to the MDRD formula by Levey *et al*.^11^. Impaired kidney function was defined as eGFR ≤60 mL/min.

The 9 mL EDTA tube was centrifuged at 2000 g for 15 minutes (15 °C) and the plasma supernatant was aliquoted and immediately frozen on dry ice and stored at −80 °C until measurement of PAPP-A2 with the PAPP-A2 ELISA kit (AL-109, Ansh Labs, TX, USA)^3^.

### Statistical analysis

We excluded participants with missing data in socio-demographic factors (n = 5), in anthropometric measurements (n = 1) and in standard laboratory measurements (n = 11). In addition, we excluded one female participant with a PAPP-A2 concentration 10-times the standard deviation (SD) over the mean. The final sample size for analysis therefore included 387 participants (157 men and 230 women).

Age in years was calculated as the difference between date of study center visit and birth date and categorized into age groups (20- < 30, 30- < 40, 40- < 50, 50- < 60, >  = 60 years). Body mass index (BMI) was calculated as weight (in kg) divided by height (in m) squared.

We analysed PAPP-A2 concentrations by sex and age. Further, age-, sex-, and pretest-adjusted mean PAPP-A2 concentrations (and 95% confidence intervals (CI)) were calculated by categories of lifestyle and medical characteristics. Correlation coefficients between PAPP-A2 concentration and age and partial correlation coefficients between PAPP-A2 concentration and anthropometric measures adjusting for age, sex and pretest were computed. Multivariable linear regression (generalized linear model) was used to evaluate the association between PAPP-A2 concentrations and anthropometric and biochemical parameters adjusted for age (continuous), pretest, weight (continuously) and sex. We calculated the coefficients from these models to reflect the estimated difference in PAPP-A2 concentration for a one unit difference in anthropometric as well as for a one standard deviation difference in the biochemical factors. We repeated the linear regression for men and women separately and calculated p-values for differences between sexes. Further, we performed separate analyses for women with menstrual cycles and women without.

In sensitivity analysis we repeated the main analyses with prior exclusion of participants with self-reported osteoporosis, diabetes, autoimmune diseases, or inflammatory bowel diseases, or impaired kidney function.

## Results

The study population comprised 387 participants (157 men, 230 women) with a mean age of 49 years (range, 20 to 70 years). BMI, smoking and alcohol consumption characteristics varied slightly between the sexes, with a higher percentage of men being overweight, obese, current smoker and consuming ≥2 alcoholic drinks/week compared to women (data not shown). Of the 230 women, none reported to be currently pregnant.

**Figure 1:**
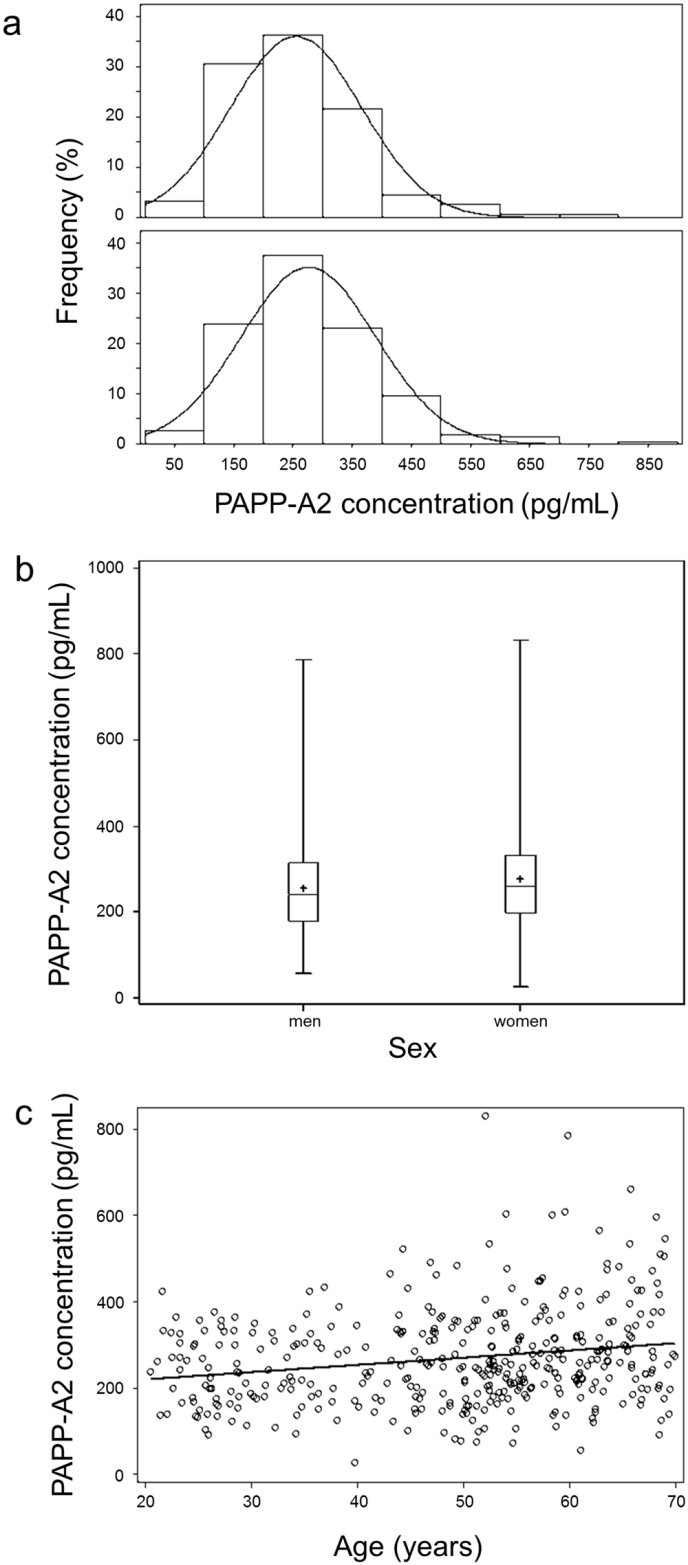
Graphical presentation of plasma PAPP-A2 concentration (n = 387), (**a**) frequency distribution (%) of PAPP-A2 concentration (pg/mL) by sex, (**b**) boxplot of PAPP-A2 concentration (pg/mL) stratified by sex, (**c**) scatterplot of PAPP-A2 concentration (pg/mL) above age (years).

PAPP-A2 concentrations were determined in plasma samples of all 387 participants with a median of 251 pg/mL ranging from 26 pg/mL to 831 pg/mL (interquartile range (IQR): 190–330 pg/mL). Graphical displays of PAPP-A2 concentrations can be found in Fig. 1. In unadjusted analyses, PAPP-A2 concentrations tended to be higher among women (median, 259 pg/mL; IQR, 199–333 pg/mL) compared to men (242 pg/mL; IQR, 179–316 pg/mL; Fig. 1b). PAPP-A2 concentrations were also positively correlated with age (Spearman correlation coefficient, r = 0.19; p < 0.001; Fig. 1c). This correlation was similar in men (r = 0.23; p < 0.005) and women (r = 0.17; p = 0.01). After adjustment for sex and pretest the correlation between PAPP-A2 concentration and age was r = 0.17 (p = 0.001).

Age-, sex-, and pretest-adjusted mean PAPP-A2 concentrations (and 95% CI) according to lifestyle and medical characteristics are given in Table 1. Similar to the unadjusted analyses, PAPP-A2 concentrations were higher in women (283 pg/mL) compared to men (261 pg/mL) when adjusted for age and pretest. Mean PAPP-A2 concentration did not differ significantly by smoking status. Participants who consumed alcohol 1–4x/month tended to have lower mean PAPP-A2 concentration than those who did not drink/only at special occasions, or those drinking ≥2x per week (p = 0.04). The proportion of participants with osteoporosis, diabetes, autoimmune diseases, inflammatory bowel diseases or impaired kidney function was low. Participants with inflammatory bowel diseases tended to have higher PAPP-A2 concentrations in comparison with participants indicating no such disease.

**Table 1:** Age-, sex- and pretest-adjusted means of PAPP-A2 concentration (pg/mL) over lifestyle and medical characteristics.

		N	%	PAPP-A2 conc. (pg/mL)		p-value^b^
				mean^a^	95% CI	
Sex						0.05
Men		157	40.6	261	(243–280)	
Women		230	59.4	283	(268–299)	
Age group						**0.03**
< 30 y		58	15.0	**241**	**(211–270)**	
30- < 40 y		45	11.6	**252**	**(218–285)**	
40- < 50 y		67	17.3	**268**	**(240–295)**	
50- < 60 y		122	31.5	**277**	**(256–298)**	
≥ 60 y		95	24.5	**298**	**(275–321)**	
Smoking status^c^						0.34
never		203	52.5	274	(257–290)	
current		85	22.0	283	(258–308)	
former		98	25.3	259	(236–282)	
Alcohol consumption^c^						0.04
never/special occ.		86	22.2	296	(272–321)	
1–4x month		165	42.6	258	(238–277)	
≥ 2x week		135	34.9	274	(253–294)	
Osteoporosis^c,d^						0.59
no		371	95.9	272	(258–285)	
yes		14	3.6	288	(229–347)	
Diabetes^d^						0.10
no		368	95.1	270	(257–283)	
yes		19	4.9	314	(263–364)	
Autoimmune diseases^c,d^						0.89
no		376	97.2	272	(259–286)	
yes		9	2.3	267	(194–340)	
Inflammatory bowel disease^c,d^						**0.03**
no		378	97.7	**271**	**(258–284)**	
yes		5	1.3	**378**	**(282–475)**	
Impaired kidney function^e^						0.45
no		372	96.1	272	(258–285)	
yes		15	3.9	294	(237–351)	

PAPP-A2, pregnancy-associated plasma protein-A2; conc., concentration; CI, confidence interval; ^a^adjusted for age (continuously), pretest and sex (where appropriate). ^b^ANOVA. ^c^Numbers do not add up to 100% because of missings. ^d^Information on diseases was self-reported by the participants. ^e^Impaired kidney function is defined as an estimated glomerular filtration rate ≤ 60 mL/min (calculated from measured serum creatinine concentration according to the MDRD formula by Levey *et al*.^11^).

With respect to the anthropometric parameters, in age-, sex- and pretest-adjusted analyses we found inverse correlations coefficients and associations of PAPP-A2 concentrations with BMI (p = 0.03) and weight (p = 0.01) (Table 2). When stratified by sex, these inverse associations were slightly stronger in women compared to men, although the difference between sexes was not statistically significant (p = 0.83 and p = 0.35, respectively). Height, waist and hip circumference were not significantly associated with PAPP-A2 concentrations at the 5%-level.

**Table 2:** Multivariable-adjusted correlations and associations between anthropometric parameters and PAPP-A2 concentration (pg/mL).

	All (n = 387)			Men (n = 157)			Women (n = 230)			p for difference
	Partial correlation coefficient^a^	Diff. in PAPP-A2 conc. (pg/mL)^b^	p-value	Partial correlation coefficient^a^	Diff. in PAPP-A2 conc. (pg/mL)^b^	p-value	Partial correlation coefficient^a^	Diff. in PAPP-A2 conc. (pg/mL)^b^	p-value	
*Per unit difference in*										
BMI (kg/m^2^)	−0.11	**−2.7**	**0.03**	−0.10	−2.4	0.21	−0.11	−2.7	0.10	0.83
Weight (kg)	−0.13	**−1.0**	**0.01**	−0.09	−0.6	0.26	−0.15	**−1.3**	**0.02**	0.35
Height (cm)	−0.06	−1.0	0.24	0.01	0.1	0.95	−0.10	−1.7	0.12	0.30
Hip circumference (cm)	−0.08	−1.0	0.12	−0.07	−0.9	0.41	−0.08	−0.9	0.24	0.97
Waist circumference (cm)	−0.09	−0.8	0.10	−0.04	−0.4	0.61	−0.11	−1.1	0.10	0.46

PAPP-A2, pregnancy-associated plasma protein-A2; diff., difference; conc., concentration; BMI, body mass index.

^a^Partial correlation coefficient between PAPP-A2 concentration and anthropometric measures while adjusting for age (continuously), pretest and sex (where appropriate). ^b^Separate linear regression models for each anthropometric parameter adjusted for age (continuously), pretest and sex (where appropriate).

The mean ( ± SD) concentrations of biochemical parameters as well as the difference in PAPP-A2 concentrations for a one standard deviation difference in concentrations of biochemical parameters is presented in Table 3. After adjustment for age, sex, weight, and pretest, we found statistically significant positive associations of PAPP-A2 concentration with GGT, ASAT and with LDH concentrations, and statistically significant inverse associations with haemoglobin concentration, haematocrit and red blood cell count (Table 3). For example, one SD higher LDH concentrations were associated with 14.9 pg/mL higher PAPP-A2 concentrations (p = 0.02), whereas one SD higher haemoglobin concentrations were associated with 19.2 pg/mL lower PAPP-A2 concentrations (p = 0.01). The remaining biomarkers were not significantly associated with PAPP-A2. In sex stratified analysis (Table 3), we found that the inverse associations of PAPP-A2 with red blood cells were restricted to women (p-value for difference in the associations between men and women, p = 0.04). Thus, in women, one SD higher red blood cell count was associated with 36.4 pg/mL lower PAPP-A2 concentrations (p = 0.0002), whereas no such difference was found in men (−9.7 pg/mL, p = 0.33). Similarly, the associations between PAPP-A2 concentration and haemoglobin concentration or haematocrit were borderline significant (p-value for difference = 0.05 and 0.07, respectively) hinting that these associations might be found in women but not in men.

**Table 3:** Mean ( ± SD) concentrations of biochemical parameters and multivariable-adjusted associations between biochemical parameters and PAPP-A2 concentration (pg/mL).

	Mean	±SD	All (n = 387)^a^		Men (n = 157)^b^		Women (n = 230)^b^		p for difference
			Diff in PAPP-A2 conc. (pg/mL)	p-value	Diff in PAPP-A2 conc. (pg/mL)	p-value	Diff in PAPP-A2 conc. (pg/mL)	p-value	
*Per SD difference*									
Creatinine	71.42	15.80 µmol/L	3.0	0.66	7.1	0.41	−4.6	0.69	0.35
Uric acid	294.14	84.35 µmol/L	0.5	0.95	−7.0	0.52	8.8	0.42	0.52
Triglycerides	1.69	1.12 mmol/L	−1.4	0.82	2.6	0.72	−10.3	0.35	0.23
HDL-C	1.57	0.50 mmol/L	1.2	0.86	8.0	0.53	−2.1	0.78	0.81
LDL-C	3.37	1.00 mmol/L	−4.4	0.49	0.0	0.99	−6.7	0.45	0.57
Cholesterol	5.37	1.15 mmol/L	−4.8	0.44	2.4	0.80	−9.8	0.26	0.38
HbA_1c_	5.62	0.45%	1.9	0.76	−1.4	0.87	4.8	0.59	0.89
Potassium	4.17	0.28 mmol/L	−1.4	0.81	5.4	0.56	−5.1	0.50	0.39
Sodium	139.46	1.96 mmol/L	8.1	0.15	4.6	0.62	10.4	0.15	0.70
GGT	0.46	0.45 µkat/L	**13.6**	**0.02**	**16.3**	**0.04**	10.0	0.28	0.52
ASAT	0.39	0.14 µkat/L	**18.5**	**0.002**	**20.6**	**0.01**	14.9	0.15	0.75
ALAT	0.41	0.25 µkat/L	11.4	0.07	13.6	0.08	4.1	0.74	0.46
LDH	2.67	0.54 µkat/L	**14.9**	**0.02**	**20.8**	**0.02**	9.4	0.32	0.27
Haemoglobin	8.34	0.72 mmol/L	**−19.2**	**0.01**	−4.7	0.70	**−33.6**	**0.002**	0.05
Haematocrit	0.40	0.03	**−17.6**	**0.01**	−5.1	0.63	**−30.5**	**0.002**	0.07
Red blood cells	4.47	0.38 Tpt/L	**−21.5**	**0.001**	−9.7	0.33	**−36.4**	**0.0002**	**0.04**
White blood cells	6.49	1.78 Gpt/L	−7.5	0.18	−15.2	0.09	−2.0	0.78	0.33

PAPP-A2, pregnancy-associated plasma protein-A2; diff., difference; conc., concentration; HDL-C, high-density lipoprotein cholesterol; LDL-C, low-density lipoprotein cholesterol; HbA_1c_, glycated haemoglobin A1; GGT, γ-glutamyl transferase; ASAT, aspartate transaminase; ALAT, alanine aminotransferase; LDH, lactate dehydrogenase.

^a^Separate linear regression models for each biochemical parameter adjusted for age (continuously), sex, weight and pretest. ^b^Separate linear regression models for each biochemical parameter adjusted for age (continuously), weight and pretest.

We decided further to stratify the analysis into women who indicated to still have menstrual cycles (n = 113) and women who did not (n = 112) (Table 4). Women with menstrual cycles had a mean age of 38 years (IQR: 27–48 years) and women without of 58 years (IQR: 54–64 years). In these analyses, we found the inverse associations between PAPP-A2 concentration and haemoglobin concentration, haematocrit or red blood cell count to be slightly stronger in women without menstrual cycles compared to women with menstrual cycles; however, tests for interaction were not significant.

**Table 4:** Multivariable-adjusted associations between distinct biochemical parameters and PAPP-A2 concentration (in pg/mL) in all women and stratified by women with and without menstrual cycles^a^.

	All women (n = 230)		Women with menstrual cycles (n = 113)		Women without menstrual cycles (n = 112)		p for difference
	Diff. in PAPP-A2 conc. (pg/mL)	p-value	Diff. in PAPP-A2 conc. (pg/mL)	p-value	Diff. in PAPP-A2 conc. (pg/mL)	p-value	
*Per SD difference in*							
Haemoglobin	**−33.6**	**0.002**	−17.7	0.16	**−46.1**	**0.007**	0.27
Haematocrit	**−30.5**	**0.002**	−15.9	0.18	**−38.8**	**0.009**	0.42
Red blood cells	**−36.4**	**0.0002**	−11.0	0.41	**−46.7**	**0.0007**	0.19

PAPP-A2, pregnancy-associated plasma protein-A2; diff., difference; conc., concentration.

^a^Separate linear regression models for each biochemical parameter adjusted for age (continuously), weight (continuously) and pretest.

Finally we repeated our main analyses with prior exclusion of participants with osteoporosis, diabetes, autoimmune diseases, inflammatory bowel diseases, or impaired kidney function (Table 5). Within this study population of 330 participants (136 men and 194 women) we found associations between PAPP-A2 concentration and anthropometric or laboratory markers not substantially different from the main analysis.

**Table 5:** Multivariable-adjusted associations between anthropometric and biochemical parameters and PAPP-A2 concentration (pg/mL) in participants without osteoporosis, diabetes, autoimmune diseases, inflammatory bowel diseases, or impaired kidney function (n = 330)^a^.

	All (n = 330)		Men (n = 136)		Women (n = 194)		p for difference
	Diff. in PAPP-A2 conc. (pg/mL)	p-value	Diff. in PAPP-A2 conc. (pg/mL)	p-value	Diff. in PAPP-A2 conc. (pg/mL)	p-value	
*Per unit difference in*							
BMI (kg/m2)	**−2.8**	**0.04**	−1.8	0.43	−3.4	0.05	0.50
Weight (kg)	**−1.0**	**0.02**	−0.5	0.42	**−1.4**	**0.02**	0.26
Height (cm)	−0.9	0.29	−0.2	0.89	−1.4	0.21	0.53
Hip circumference (cm)	−0.9	0.18	−0.3	0.82	−1.1	0.17	0.54
Waist circumference (cm)	−1.0	0.06	−0.1	0.93	**−1.7**	**0.02**	0.15
*Per SD difference in*							
Creatinine	−6.7	0.39	0.9	0.94	−13.8	0.22	0.30
Uric acid	−3.7	0.65	−5.8	0.64	−0.1	0.99	0.98
Triglycerides	−0.6	0.93	2.6	0.74	−8.7	0.46	0.26
HDL-C	2.7	0.69	13.7	0.33	−2.2	0.78	0.59
LDL-C	−1.0	0.89	2.3	0.82	−3.6	0.72	0.53
Cholesterol	−0.9	0.90	4.6	0.64	−6.3	0.52	0.39
HbA_1c_	−0.8	0.91	−3.4	0.73	1.6	0.87	0.93
Potassium	3.9	0.53	12.0	0.24	−0.1	0.99	0.35
Sodium	8.4	0.16	1.7	0.86	13.4	0.09	0.37
GGT	10.5	0.09	**21.0**	**0.02**	−2.1	0.82	0.05
ASAT	**19.0**	**0.003**	**25.7**	**0.004**	8.9	0.36	0.22
ALAT	10.9	0.11	16.8	0.06	−4.1	0.73	0.12
LDH	**16.7**	**0.01**	**23.1**	**0.02**	9.3	0.35	0.21
Haemoglobin	**−19.5**	**0.02**	−1.2	0.93	**−36.1**	**0.001**	**0.03**
Haematocrit	**−19.4**	**0.01**	−2.0	0.88	**−35.3**	**0.0006**	**0.03**
Red blood cells	**−20.0**	**0.006**	−8.7	0.42	**−35.6**	**0.0009**	0.05
White blood cells	**−12.0**	**0.04**	−18.1	0.06	−7.1	0.36	0.44

PAPP-A2, pregnancy-associated plasma protein-A2; diff., difference; conc., concentration.

^a^Separate linear regression models for each anthropometric or biochemical parameter adjusted for age (continuously), pretest and where appropriate for sex and weight (continuous).

## Discussion

In this cross-sectional study we measured plasma PAPP-A2 concentrations in adult men and non-pregnant women of a general study population. The PAPP-A2 concentrations tended to be higher in women in comparison to men, and positively correlated with age. While smoking status was not associated with PAPP-A2 concentration, we found weak associations with body weight, BMI and alcohol consumption. Among biochemical factors, GGT, ASAT and LDH concentrations were positively associated with PAPP-A2 concentration. Haemoglobin concentration, haematocrit and red blood cell count were inversely associated with PAPP-A2, although interaction analyses showed that the inverse associations were restricted to women only.

PAPP-A2 is highly expressed during the course of pregnancy, with a potential role in foetal growth and a potential prognostic value for evaluating pre-eclampsia^3, 6, 7^. Here we present the first study measuring PAPP-A2 concentration in a large population independent of the pregnancy context. In 2013, Kløverpris *et al*. introduced an immunoassay for PAPP-A2 measurement in human serum^3^. During assay testing, they measured PAPP-A2 concentration in serum samples of two male and two female participants and reported a mean concentration of 300–500 pg/mL^3^, which is in line with our findings using the same immunoassay.

This far, research has pointed to a role of PAPP-A2 in foetal growth and development, since it is highly expressed in the placenta during pregnancy and supposed to increase IGF bioavailability by cleavage of binding proteins IGFPB-3 and IGFPB-5^2^. Furthermore, members of two families were recently identified, whose homozygous mutations in *PAPPA2* (p.D643fs25* and p.Ala1033Val), lead to a complete absence of PAPP-A2 proteolytic activity resulting in increased IGF-1 bound to IGFBPs and decreased free IGF-1 concentrations. The affected individuals were characterized by progressive growth failure, moderate microcephaly, thin long bones and mildly decreased bone density^9^. In our analysis in an adult population, we did not find an association between height and PAPP-A2 plasma concentration. However, it is quite reasonable, that the potential causal relationships between PAPP-A2 concentration and growth do not persist into adulthood, when the growth period is over.

With respect to the other anthropometric measures, we found indications for weak inverse associations of PAPP-A2 concentration with weight and BMI. From a theoretical perspective, IGF-1 is supposed to be central to the regulation of anabolic (growth) processes^12^; however, the current knowledge of the biological function of PAPP-A2 is too scarce to draw inferences. Much more complexity is added since the relationship between BMI and IGF-1 levels is presumably inversely U-shaped, while free IGF increases with obesity^13^. Furthermore, elevated serum IGF-1 levels have been found to be associated with reduced risk of osteoporosis^14^ and diabetes^15^ on the one hand, while they are associated with a higher risk of breast, colorectal, prostate and lung cancer^16, 17^ on the other. Thus, to elucidate the role of PAPP-A2 in an adult population, a next step would be to determine the relationship of PAPP-A2 with concentrations of IGF-1, free IGF1 and IGF binding proteins in an adult population.

We had self-reported information on diagnoses of diabetes, osteoporosis, autoimmune diseases and inflammatory bowel diseases in this study population. We found higher adjusted mean PAPP-A2 concentrations in participants reporting to have inflammatory bowel diseases in comparison to participants without this condition. Furthermore the adjusted means PAPP-A2 concentration in participants indicating diabetes was slightly although not significantly higher than in participants without diabetes. It should be noted that the self-reported diagnoses of diseases in this study were not validated by physicians’ records or medication information. In addition, given that our study population was selected from the general population, the number of diseased persons was relatively small. Therefore, results for these subgroups need to be interpreted cautiously. Future studies are warranted to explore the association of PAPP-A2 concentration with respect to diabetes or inflammatory bowel diseases.

With respect to biochemical parameters, we found a positive association between PAPP-A2 concentration and GGT, ASAT and LDH serum concentration. We point out, that in this study population the concentration of all three parameters varied within the normal clinical range, which is also true for white blood cell count as a standard marker for inflammation. Since all three parameters might function as chronic markers of cell damage^18–20^ irrespective of an acute inflammatory event, one might speculate, that in the same way as GGT, ASAT or LDH are released from cytosol to the circulation upon cell damage, PAPP-A2 is released, thus leading to increased plasma concentrations.

Interestingly, we found an inverse association between PAPP-A2 concentration and concentrations of haemoglobin, haematocrit and red blood cells, which was restricted to women. Some studies suggest that concentrations of haemoglobin may fluctuate according to the menstrual cycle, due to menstrual blood loss^21^. One may therefore speculate that the inverse association with these blood parameters indicates that PAPP-A2 concentrations also depend on menstrual cycle. However, when we stratified the analysis into women still having menstrual cycles and those who have not we surprisingly found that the inverse association was even stronger among women not having menstrual cycles and weaker (and statistically non-significant) among women having menstrual cycles, although the difference between both groups of women was statistically not significant.

The strengths of our study include the selection of the study population, which was predominantly drawn from the general population through population registries. It should be noted, subjects willing to participate in medical studies are likely to be more health conscious than others, and, therefore our study may not be representative for the general population^22^. Nevertheless, the wide variability with respect to age, body mass and lifestyle characteristics allowed us to demonstrate association between PAPP-A2 concentration and several parameters which might be generalizable. The size of our populations seems appropriate to show biological meaningful differences in PAPP-A2 concentrations over various lifestyle, anthropometric or biochemical parameters and to repeat analyses stratified by gender. Data assessment followed standardized procedures and was conducted by trained personnel, especially the measurement of anthropometric parameters followed the WHO protocol^23^ and did not rely on self-reports, which are prone to some errors^24^. Furthermore all clinical laboratory measurements were performed in the same laboratory following standardized procedures and within 6 hours from blood drawing. However, triglyceride concentration may be affected by fasting status^25^ and fasting was not a prerequisite in our study; thus, the results with respect to triglycerides concentration should be interpreted cautiously. Measurement of LDL-C was performed directly, and thus do not depend on fasting status^26^. In addition, the method used to quantify PAPP-A2 concentration has been extensively tested to determine limit of detection, linearity of dilution and spike recovery^3^. However, the cross-sectional design of our study limits causal inferences.

In conclusion, this study presented PAPP-A2 concentrations measured in a general adult population of men and non-pregnant women. PAPP-A2 concentration was measurable in all participants which is a prerequisite for all future studies aiming to further elucidate the biological role of PAPP-A2 in adults. We were able to show a widespread distribution of PAPP-A2 concentration with variation over age, sex and weight. PAPP-A2 concentrations were positively associated with age and serum concentration of GGT, ASAT and LDH and negatively associated with weight in all participants and with serum concentration of haemoglobin, haematocrit and red blood cell count in women. However, it is important to direct future research to the simultaneous measurement of IGF-1 and IGFBPs, since these are the primary targets of the metalloproteinase PAPP-A2.

### Data availability

The datasets generated during and/or analysed during the current study are not publicly available due to participants’ data protection reasons and the informed consent, which does not foresee public depositing; but datasets are accessible from the corresponding author on reasonable request.
